# Evolutionary history and current distribution of the West Mediterranean lineage of *Brucella melitensis* in Italy

**DOI:** 10.1099/mgen.0.000446

**Published:** 2020-10-08

**Authors:** Anna Janowicz, Fabrizio De Massis, Katiuscia Zilli, Massimo Ancora, Manuela Tittarelli, Flavio Sacchini, Elisabetta Di Giannatale, Jason W. Sahl, Jeffrey T. Foster, Giuliano Garofolo

**Affiliations:** ^1^​ National and OIE Reference Laboratory for Brucellosis, Istituto Zooprofilattico Sperimentale dell’Abruzzo e del Molise "G. Caporale", via Campo Boario, 64100 Teramo, Italy; ^2^​ Pathogen and Microbiome Institute, Northern Arizona University, Flagstaff, AZ 86011, USA

**Keywords:** *Brucella melitensis*, brucellosis, molecular epidemiology, WGS

## Abstract

Ovine and caprine brucellosis, caused by *Brucella melitensis *, is one of the world’s most widespread zoonoses and is a major cause of economic losses in domestic ruminant production. In Italy, the disease remains endemic in several southern provinces, despite an ongoing brucellosis eradication programme. In this study, we used whole-genome sequencing to detail the genetic diversity of circulating strains, and to examine the origins of the predominant sub-lineages of *B. melitensis* in Italy. We reconstructed a global phylogeny of *B. melitensis *, strengthened by 339 new whole-genome sequences, from Italian isolates collected from 2011 to 2018 as part of a national livestock surveillance programme. All Italian strains belonged to the West Mediterranean lineage, which further divided into two major clades that diverged roughly between the 5th and 7th centuries. We observed that Sicily serves as a brucellosis burden hotspot, giving rise to several distinct sub-lineages. More than 20 putative outbreak clusters of ovine and caprine brucellosis were identified, several of which persisted over the 8 year survey period despite an aggressive brucellosis eradication campaign. While the outbreaks in Central and Northern Italy were generally associated with introductions of single clones of *B. melitensis* and their subsequent dissemination within neighbouring territories, we observed weak geographical segregation of genotypes in the southern regions. Biovar determination, recommended in routine analysis of all *Brucella* strains by the World Organisation for Animal Health (OIE), could not discriminate among the four main global clades. This demonstrates a need for updating the guidelines used for monitoring *B. melitensis* transmission and spread, both at the national and international level, and to include whole-genome-based typing as the principal method for identification and tracing of brucellosis outbreaks.

## Data Summary

Sequence reads for all 339 isolates were deposited in the Sequence Read Archive (SRA) under BioProject accession number PRJNA615379. A full list of SRA sample accession numbers and the associated metadata are provided in Table S1 (available with the online version of this article).

**Impact Statement**

Ovine and caprine brucellosis is a major zoonotic disease transmitted to humans most commonly via contaminated milk or dairy products. In our study, we sequenced and analysed 339 Italian strains of *Brucella melitensis *; therefore, increasing the publicly available genomes belonging to the West Mediterranean lineage, which to date has been under-represented. We established a possible period of diversification of *B. melitensis* clades in Italy more than a millennium ago, and described the recent epidemiology of ovine and caprine brucellosis in Italy over a period of 8 years. We demonstrated the use of whole-genome-sequencing methods to monitor the spread of clonal complexes at the national level, which revealed that the eradication campaign implemented in Italy has not been effective in eliminating some of the circulating clones, especially in the regions with the highest prevalence of brucellosis. Such hotspots of infection must, therefore, be considered as high-priority targets in order to eliminate the risk of brucellosis transmission to the other regions.

## Introduction

*Brucella melitensis* is the causative agent of ovine and caprine brucellosis, and it is the most common cause of brucellosis in humans [1–3]⁠. Small ruminants, including sheep and goats, are the natural hosts, but *B. melitensis* can also infect other animals, such as cattle (*Bos taurus*), water buffalo (*Bubalus bubalis*) and camels (*Camelus* spp.) [4–6]⁠. Brucellosis is among the most widespread zoonotic diseases worldwide, yet it is still considered a neglected zoonosis due to the limited resources devoted to its surveillance and control, particularly in economically developing countries [7–9]⁠. Although infection with *B. melitensis* in humans is rarely fatal, it remains a major public-health concern, and in misdiagnosed and untreated patients it can progress to a chronic and debilitating disease with severe complications [10–12]⁠.

Ovine and caprine brucellosis likely first established in the Fertile Crescent, where wild goats and sheep were first domesticated [13–16]⁠. From there, three main *B. melitensis* clades developed: an Americas group that includes strains from Spain, Portugal and much of North and South America, an East Mediterranean lineage that extends from the Middle East across to East Asia, and a West Mediterranean lineage found largely in Italy and the Maghreb [17–20]⁠. An additional lineage, the African clade, diverged from the Americas group, and is found primarily in East Africa and the Arabian Peninsula [21]⁠. Ovine and caprine brucellosis have now been eliminated from much of the Western world, but it is still present in domestic and wild animals in the Mediterranean Basin, Western Asia, and parts of Africa and South America, with re-emergence in the Middle East [22–24]⁠. In the European Union (EU), the disease remains present in small ruminant flocks in several countries, including Croatia, Greece, Italy, Spain and Portugal [25].

Ewes and does infected with brucellosis suffer from reproductive failures, while male ruminants present with orchitis, epididymitis and infertility. Aborted foetuses, placentas and mucosal secretions, as well as milk, of chronically infected animals are the main transmission sources of the disease within flocks and to humans [26, 27]⁠. Outbreaks of brucellosis in farms pose a risk to human health, and can lead to major economic losses for farmers and livestock breeders [28, 29]⁠. Thus, in order to eliminate ovine and caprine brucellosis, Italy adopted a strict eradication programme in 1992, based on a test and slaughter policy [30, 31]⁠. Since the introduction of the national plan, a slow but steady decrease in disease prevalence was observed [32]⁠. In the 20 years from 1997 to 2017, brucellosis-positive farms declined from 3.9 to 1.0%, and several northern regions were declared ‘officially free from *B. melitensis *’ [30, 33]⁠. Unfortunately, despite the surveillance and control measures introduced in the campaign, the disease still remains endemic in several southern provinces of Italy [19, 32–34]. Annually, the highest prevalence in sheep and goats is observed in Sicily, with 7604 animals in 208 flocks testing seropositive to *B. melitensis* in 2018 [35]. In the year 2016, an outbreak of human brucellosis was traced back to consumption of local soft cheese in Messina, during which 137 cases were reported; thus, highlighting the need for increased efforts to break transmission chains, especially in the most affected areas of the country [36]⁠.

In this work, we used whole-genome sequencing (WGS) to characterize *B. melitensis* strains circulating in Italy from 2011 to 2018 and to place sequenced genomes within a global phylogeny. Our approach used Bayesian temporal analysis to understand the evolutionary history and timing of the West Mediterranean lineage in Italy. Additionally, we used the computationally simplified method core-genome multilocus sequence typing (cgMLST) to determine the diversity and epidemiological relatedness of the current Italian *B. melitensis *. For surveillance purposes, data clustering using minimum spanning trees (MSTs) with the established cut-offs was performed to allow contact tracing of the outbreaks.

## Methods

### ***B. melitensis*** isolate sequences

We sequenced 339 Italian *B. melitensis* isolates for this study; sequencing reads were deposited in the National Center for Biotechnology Information (NCBI) repository under BioProject accession number PRJNA615379. The dataset was supplemented by published sequences of 102 Italian strains [37] and 88 strains collected in Sweden [38]⁠. An additional 149 genomes of *B. melitensis* with a reported isolation date were downloaded from the NCBI (accessed May 17 2019). The list of samples and the associated metadata are shown in Table S1.

### Isolation of *B. melitensis*

Animal samples were collected after slaughtering livestock found positive for brucellosis in the context of the national eradication campaign from 2011 to 2018. Lymphatic glands, uterus or udder, and spleen samples were collected during necropsy. The isolates were cultured and identified by the *Istituti Zooprofilattici Sperimentali* laboratories using the standard protocol described in the World Organisation for Animal Health (OIE) Manual of Diagnostic Tests and Vaccines for Terrestrial Animals [4]⁠. Human *B. melitensis* isolates were obtained from the blood samples of patients suspected of suffering from brucellosis. For DNA extraction, all of the *B. melitensis* isolates were sub-cultured in Brucella medium base and incubated in a 5–10 % CO_2_ atmosphere at 37 °C for 48 h to assess the purity of cultures and the absence of dissociation. DNA extraction was performed using a Maxwell 16 tissue DNA purification kit (Promega), following the manufacturer’s instructions. The isolates were identified as *B. melitensis* by PCR and biovar designation was obtained by standard biotyping as recommended by the OIE [4, 6]⁠.

### WGS

Total genomic DNA from the 339 samples was sequenced using the Illumina NextSeq 500 platform. Briefly, following total DNA quantification (Qubit DNA HS assay; Thermo Fisher Scientific), the sequencing libraries were generated using a Nextera XT library preparation kit (Illumina), according to the manufacturer’s instructions. The libraries were sequenced using NextSeq 500/550 mid output reagent cartridge v2 (300 cycles). Paired-end 150 bp reads were generated and their quality was assessed with FastQC [39]⁠. Reads were trimmed with Trimmomatic version 0.36 and subsequently used to assemble genome scaffolds using SPAdes version 3.11.1 [40, 41].

### Multilocus sequence typing (MLST) and cgMLST

Genome assemblies of Italian *B. melitensis* were genotyped using cgMLST. We used cgMLST because the method generates data that are relatively quick and simple to analyse, and the results can be readily standardized and reported. The cgMLST profiles were assigned using the *B. melitensis* task template with 2704 target core genes in Ridom SeqSphere+ software, version 4.1.1 (Ridom), as previously described [37]⁠. Only genomes containing ≥98 % of good targets were accepted for the subsequent analyses. To be accepted as good targets, identified genes need to fulfil the software’s default Target QC parameters, i.e. the same length as the reference gene ± 3 nucleotide triplets, no ambiguities and no frameshifts. A MST was constructed by pairwise comparison of cgMLST alleles. Based on previous data, the cut-off of six allele differences was applied to identify clusters of possibly related genomes [37]. Default parameters were used and the missing values were excluded in the calculation of distance between genotypes. The public genome dataset and the Italian strains sequenced in this study were genotyped with the *Brucella* nine locus MLST (MLST-9) scheme available at https://pubmlst.org/brucella/ [42]⁠ and accessed through Ridom SeqSphere+.

### SNP detection

SNPs were called using In Silico Genotyper (isg) (version 0.16.10–3), with bwa-mem (version 0.712-r1039) as the aligner and gatk (version 3.9) as the SNP caller using *B. melitensis* strain 16M (GenBank accession numbers NC_003317.1 and NC_003318.1) as the reference [43–45]. Default filters were applied to remove SNPs from duplicated regions, minimum quality was set to Phred 30 and the minimum allele frequency was set to 90 %. The alignments of concatenated, clean and unique variants were used in further analyses.

### Phylogenetic analysis

A maximum-likelihood (ML) tree was built from the sequence (*n*=499) alignment of 14 531 concatenated SNPs called by isg. RAxML-NG version 0.9.0 was used to perform the analysis with the generalized time-reversible model with gamma distribution and base frequencies optimized by ML [46]⁠. The number of invariant sites was accounted for in the model and Stamatakis correction was used to correct for ascertainment bias. Branch support for the phylogeny was assessed using the transfer bootstrap expectation method with 650 bootstrap replicates (number of sufficient bootstrap replicates was estimated by a majority rules extended (MRE) based bootstrapping test performed in RAxML-NG. The best tree was mid-point rooted and visualized using version 4.4.2 of the Interactive Tree of Life (iTOl) online tool [47]. Population structure was assessed using BAPS 6.0 [in the module hierarchical BAPS (hierBAPS)] [48]⁠.

### Temporal analysis

Divergence times for *B. melitensis* strains were estimated using the Bayesian Markov chain Monte Carlo method implemented in the program beast version 2.6.1 [49]. We first subsampled our dataset to reduce redundancy of the over-represented clades and, therefore, minimize sampling bias. The dataset of 259 samples was obtained by pruning the ML phylogenetic tree to 0.90 relative length with Treemmer [50]. The analysis was performed using an alignment of concatenated SNPs obtained using the isg tool. Two molecular clock models, strict and relaxed, were tested in combination with coalescent constant, coalescent exponential and Bayesian skyline demographic models using stepping stone/path sampling analysis in beast 2 (beast Path Sampler in Model Selection package, version 1.5.2). The beast 2 package bModelTest version 1.2.1 was used to select the optimal base substitution model in all of the analyses [51]⁠.

The strict clock model with the coalescent exponential demographic model best fit for our dataset, as previously shown for *B. melitensis* [16]. Three independent Markov chain Monte Carlo analyses were performed, each with a chain length of 200 million generations. Invariant site values were provided manually in the xml Beauti file. The first 40 million chains were discarded as a burn-in and posterior distributions were sampled every 20 000 generations. Log files were inspected using Tracer version 1.7.1 to ensure convergence and effective sample size above 200. The log and tree files of the three runs were combined using LogCombiner version 2.6.1 (Prerelease). Maximum clade creditability trees were generated using TreeAnnotator version 2.6.1 (Prerelease) and visualized using FigTree version 1.4.1.

## Results

### Global phylogeny of *B. melitensis*

**Fig. 1.**
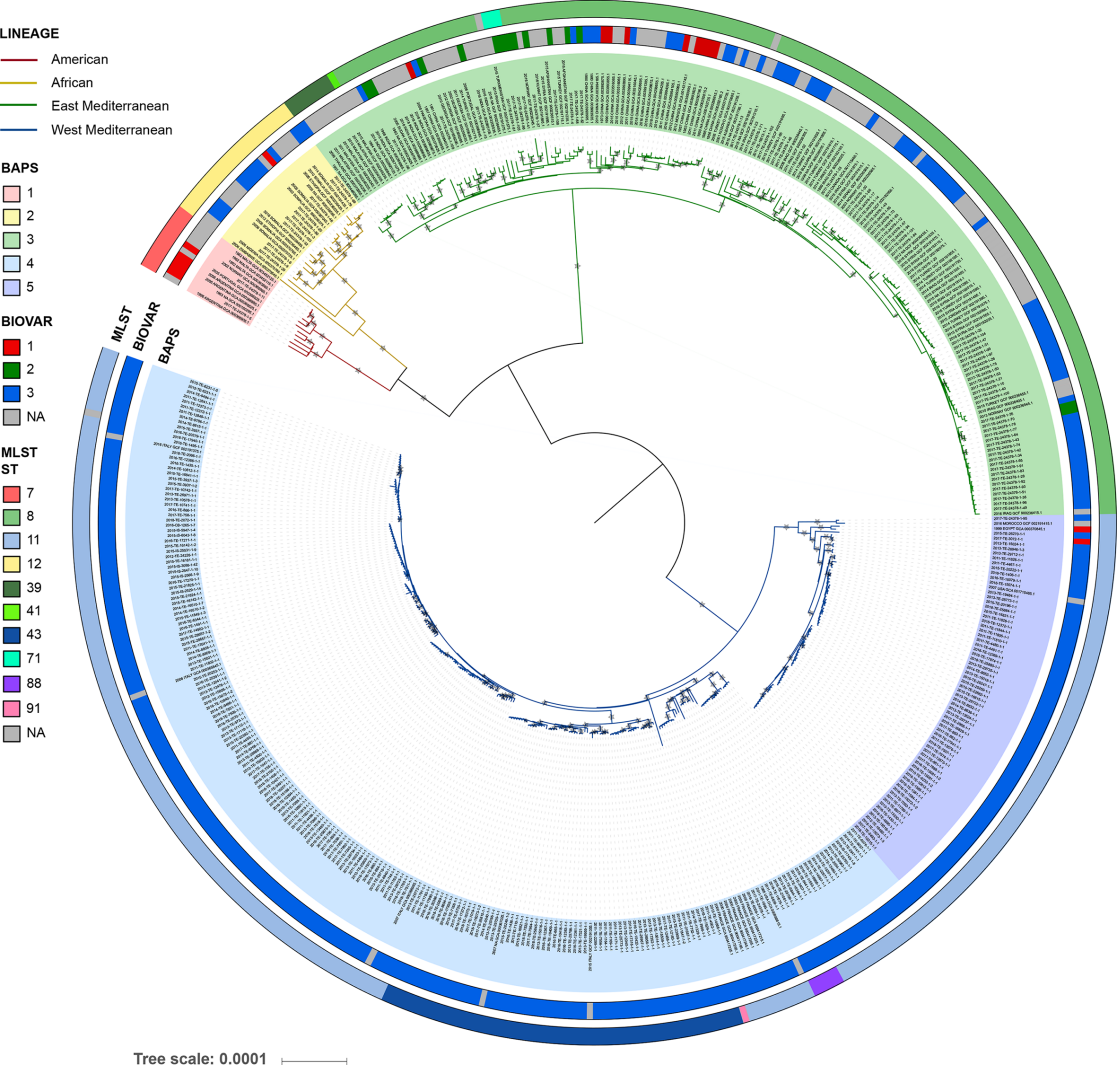
Global phylogeny of *B. melitensis *. The ML tree was generated from an alignment of 15 041 concatenated SNPs from 499 *B. melitensis* genomes. The branch colours correspond to the four main lineages and the strain ID labels are shaded according to the assigned BAPS population. The two outside rings correspond to biovar and MLST genotype. The tree is mid-point rooted, and branches with bootstrap values between 0.99 and 1 are marked with a star. Bar, mean number of nucleotide substitutions per site.

Until recently, only a few Italian *B. melitensis* genomes were publicly available, so the structure of this lineage remained poorly known. Our sequencing of 339 strains enabled a detailed reconstruction of the placement of Italian strains within a global phylogeny (Fig. 1). We identified 15 041 SNPs and used them to build a phylogenetic tree using ML. The characteristic topology of the *B. melitensis* phylogeny was observed, with Italian strains clustered within the West Mediterranean lineage, and three other phylogeographical lineages evident (Americas, African and East Mediterranean). We discovered 1004 SNPs unique to the West Mediterranean clade. Two main branches were identified within this lineage, one containing strains from Egypt, Italy and Morocco, and the other with strains from France, Italy and the USA.

**Fig. 2.**
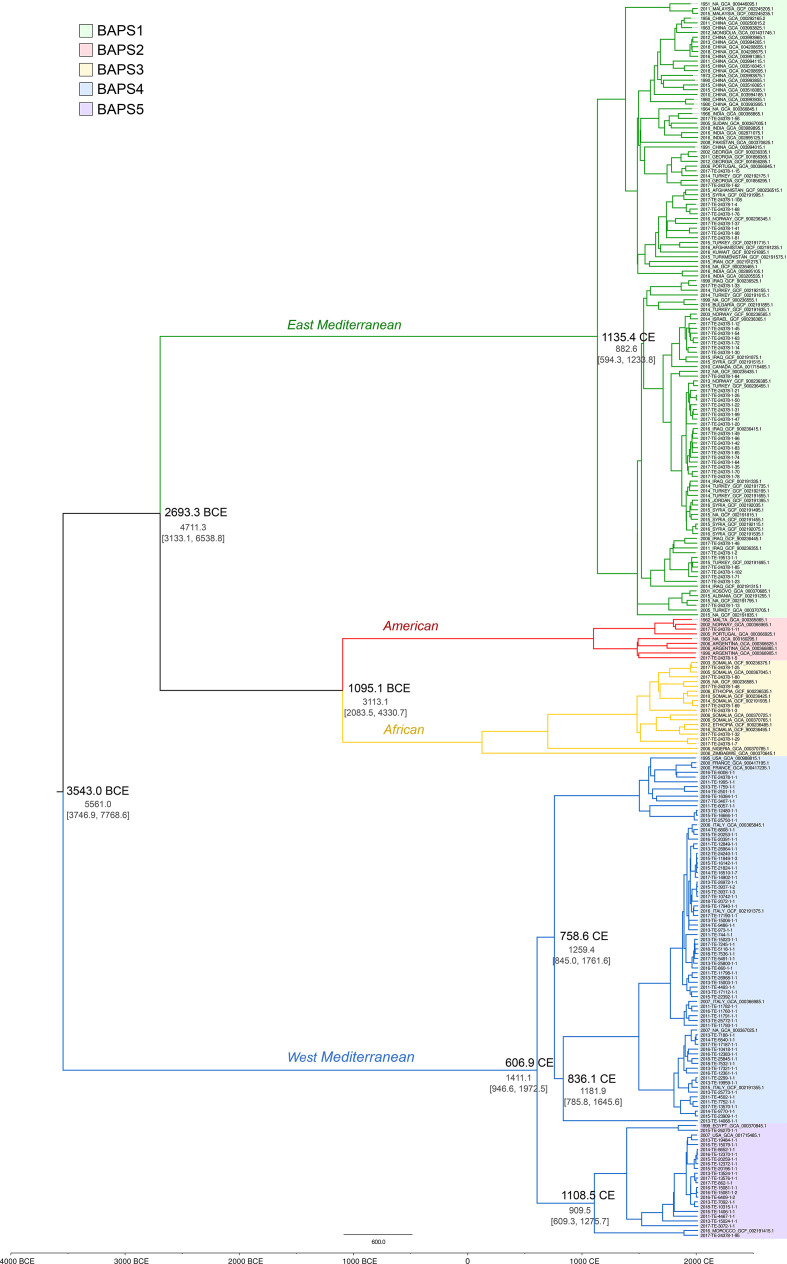
Time-structured phylogeny of a subset of 259 isolates of *B. melitensis *. The maximum clade creditability tree was built using concatenated SNP sequences. The mean node ages are shown for the major nodes together with the node heights and corresponding 95 % highest posterior density (HPD) intervals (in grey). The branches are coloured according to the lineage and the BAPS populations are highlighted with different colours as shown in the legend.

Based on a secondary level of clustering of the whole-genome SNPs, a BAPS analysis split the tree into five lineages and confirmed the presence of two diverse groups within the West Mediterranean lineage (BAPS 4 and BAPS 5) (Fig. 2). The larger clade, BAPS 4, further divided into two subpopulations, one containing only Italian strains, and the other including strains from Italy and France. All Italian *B. melitensis* isolates belonged to biovar 3, which was the most common biovar in our dataset, and was also assigned to a majority of strains from Africa and to a large subpopulation within the East Mediterranean lineage. Unfortunately, the majority of publicly available genomes lack biotyping results, so the relationship of biovar to genetic group could not be determined.

MLST analysis revealed four sequence types (STs) of *B. melitensis* present in Italy. The majority of the strains belonged to ST-11 and ST-43. ST-88 was generally confined to animals from the Apulia (Puglia) region and, interestingly, ST-91 was identified in only one isolate, which had been collected from a water buffalo in Campania. The latter isolate did not appear to belong to any sub-cluster, but formed a single long branch within the BAPS 4 population and had 270 unique SNPs.

### Origins and divergence of the West Mediterranean lineage of *B. melitensis*

According to the results of our temporal analysis, the West Mediterranean lineage was the first to diverge from the *B. melitensis* population, dividing into two branches in ~3500 BCE at the dawn of the Neolithic period, and after the first introductions of domesticated sheep and goats occurred in the Central Mediterranean region. The lineage split into two, which corresponded to BAPS 4 and BAPS 5 subclades, that shared a most recent common ancestor (MRCA) around 1400 years ago (95 % confidence interval: 946.6–1972.5). Another major division appears to have occurred less than 200 years later, which resulted in division of BAPS 4 into two subpopulations. One of these was entirely composed of Italian isolates, while the other was shared with French strains.

Emergence of a distinct branch corresponding to the *B. melitensis* isolate from a water buffalo (2013-TE-14068-1-1) occurred around the same time as the main split of BAPS 4. The temporal evolution of the genetic clade corresponding to BAPS 5 involved two divergence events, the first occurring in approximately 1100 CE and the second roughly 200 years later. Both involved diversification of Italian strains from strains found in Africa.

### Genomic epidemiology of ovine and caprine brucellosis in Italy

**Fig. 3.**
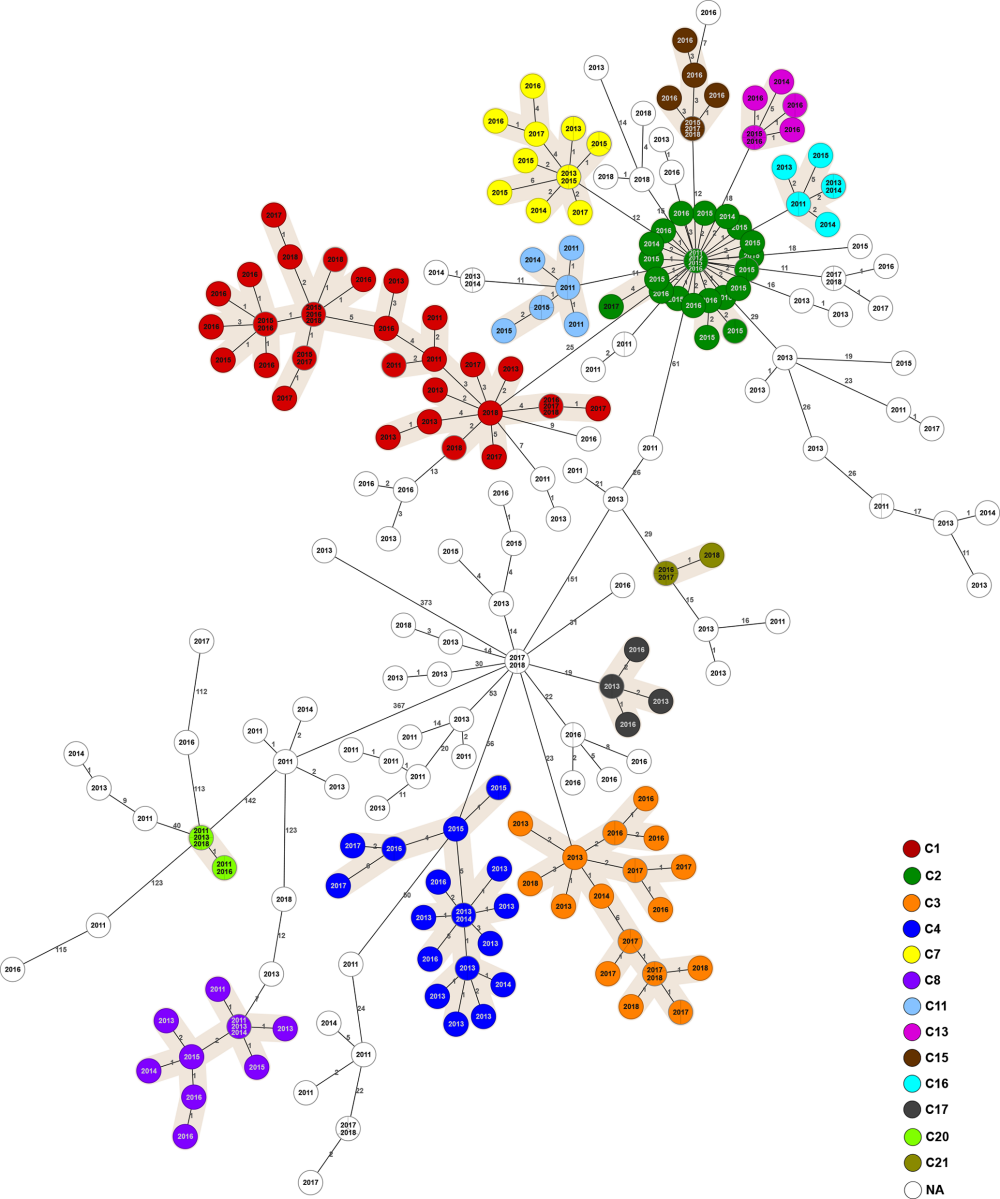
MST generated for 317 Italian isolates of *B. melitensis* in the BAPS 4 population using the gene-by-gene approach. The MST was calculated by pairwise comparison of 2704 target genes with missing values ignored. Node labels correspond to strain isolation year and the branches to the number of discriminating loci. Complexes of genotypes within a distance of six alleles and containing a minimum of five isolates are numbered and depicted with different colours. Two main phylogenetic clusters are highlighted.

**Fig. 4.**
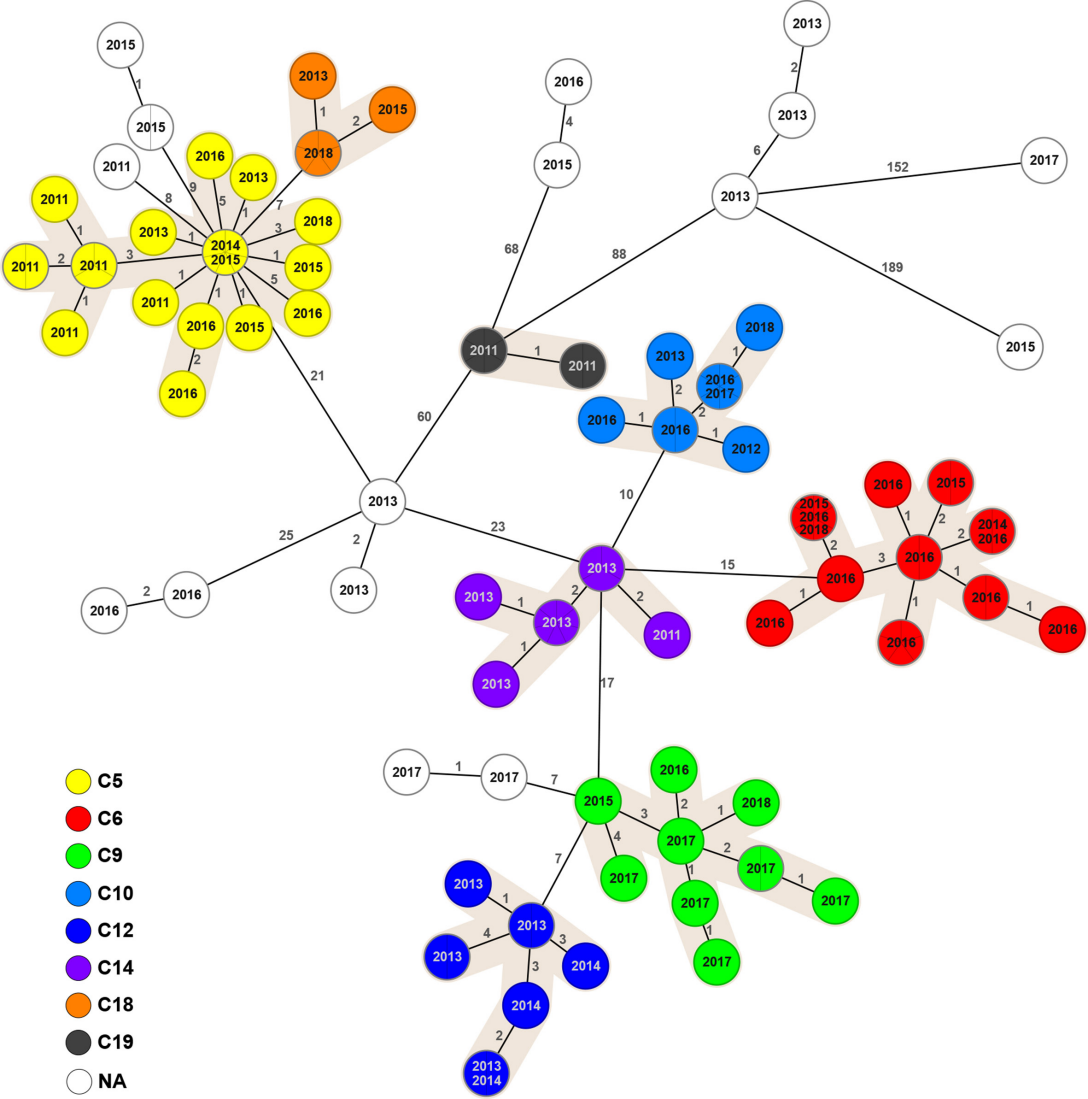
MST generated for 123 Italian isolates of *B. melitensis* in the BAPS 5 population using the gene-by-gene approach. The MST was calculated by pairwise comparison of 2704 target genes with missing values ignored. Node labels correspond to strain isolation year and the branches to the number of discriminating loci. Complexes of genotypes within a distance of six alleles and containing a minimum of five isolates are numbered and depicted with different colours. Two main phylogenetic clusters are highlighted.

The majority of *B. melitensis* strains analysed in our study were isolated from animals in Sicily (*n*=240) and Calabria (*n*=87). In particular, the territories surrounding the Strait of Messina, which joins the two regions, had a particularly high burden of brucellosis (Table S1). Epidemiological investigation using cgMLST allowed us to estimate genetic relationships among the isolates within our dataset. The strains in the two BAPS populations present in Italy differed by more than 467 allelic differences. The larger clade, BAPS 4, contained isolates collected in 13 regions of Italy (Fig. 3), while BABS 5 strains were found only in Sicily and Calabria (Fig. 4). Two subclades within the BAPS 4 population were separated by a distance of 367 allelic differences. The only strain assigned to ST-91 isolated from buffalo in 2014 differed by 372 alleles from the closest neighbouring strain, again highlighting its uniqueness.

**Fig. 5.**
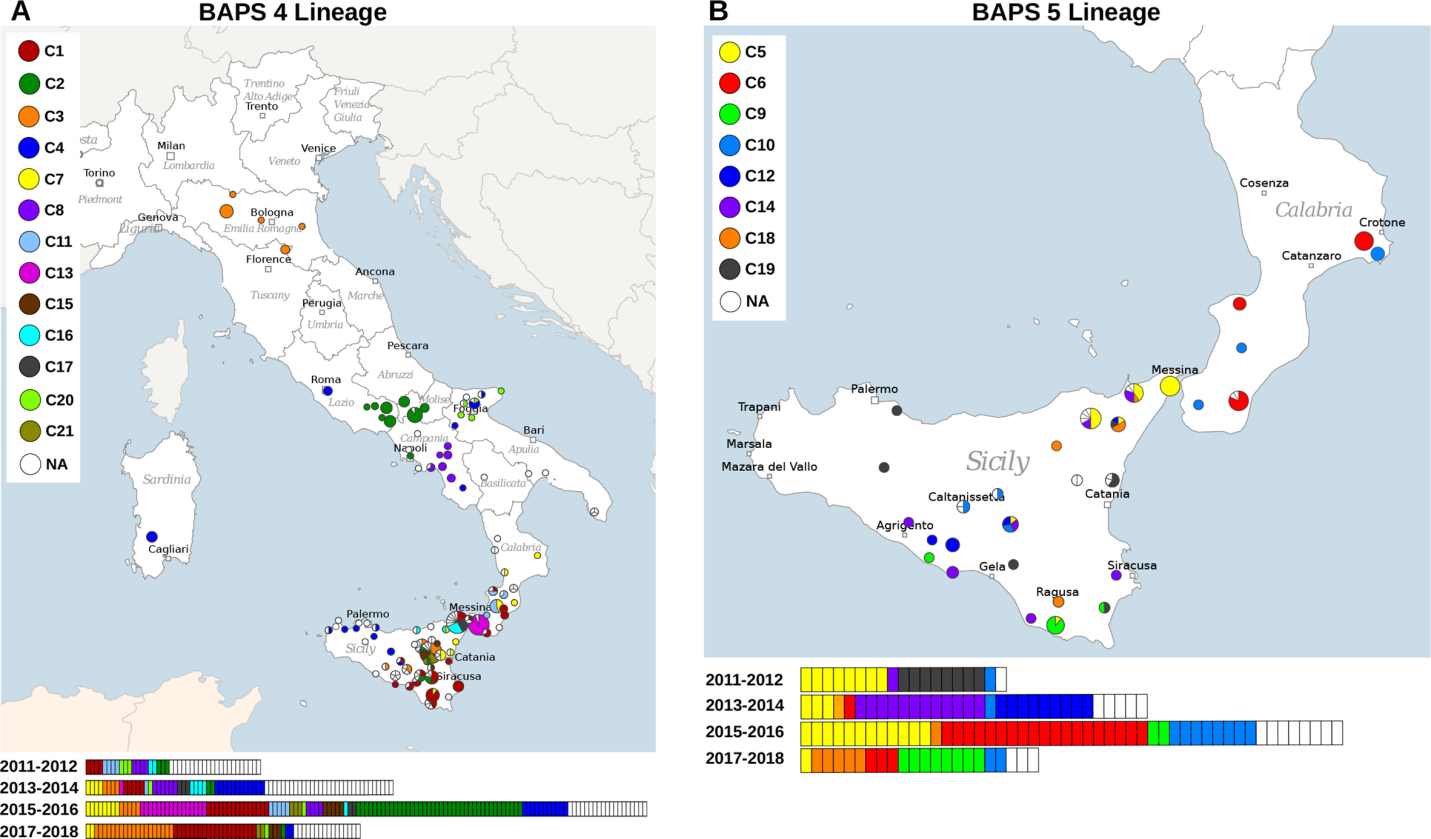
Geographical distribution of cgMLST complexes of *B. melitensis* in Italy. A set of 440 isolates was typed using cgMLST and the clusters of at least five genotypes placed within six gene distance from the nearest neighbour were grouped into complexes. The isolates were assigned to BAPS populations using hierBAPS. The BAPS 4 strains are shown in (a) and the BAPS 5 strains are shown in (b). The isolates are coloured according to the assigned complex, and the numbers of individual samples coloured according to the assigned complex and isolated in specified time period are shown under the maps.

We identified 21 cgMLST complexes containing a minimum of five strains and located within a maximum distance of six diverse core genes to the nearest neighbour (Table 1). Isolates assigned to these clusters were distributed across Italy, while the ‘sporadic cases’, i.e. not belonging to any complex, were found mainly in Sicily and Calabria. While these two regions, and Sicily in particular, are a hotspot of brucellosis, a clear geographical separation of the clonal clusters was observed in central and northern regions (Fig. 5). Epidemiological metadata suggests that some of the complexes might have originated in Sicily and then, likely due to the livestock trade, established in other parts of the country, resulting in local outbreaks of brucellosis. For example, we found several isolates from C2 that were collected in 2011 and 2012 in Sicily and subsequently, in 2015 and 2016, the same clone was identified in an outbreak in Lazio and Molise. A similar scenario was observed for C4, present in Sicily in 2013 and 2014, which in later years also emerged in Lazio, Apulia and Campania. Conversely, few complexes, such as C8 and C20, contained only strains from mainland Italy.

**Table 1.** *
B. melitensis* cgMLST complexes present in Italy between 2011 and 2018

Complex	No. of isolates	No. of genotypes	No. of farms	2011/2012	2013/2014	2015/2016	2017/2018	Host	Region	Maximum distance cgMLST
1	44	22	31	x	x	x	x	Cattle, goat, sheep	Calabria, Sicily	17
2	46	21	24	x	x	x	x	Cattle, goat, human, sheep	Abruzzi, Lazio, Molise, Sardinia, Sicily	8
3	28	17	13			x	x	Cattle, goat, sheep	Emilia Romagna, Sicily, Tuscany	12
4	25	17	19		x	x		Cattle, goat, sheep	Campania, Lazio, Apulia, Sicily	19
5	24	15	14	x	x	x	x	Cattle, goat, sheep	Sicily	10
6	23	10	7		x	x	x	Goat, sheep	Calabria	6
7	14	10	9		x	x	x	Cattle, goat, sheep	Calabria, Sicily	11
8	14	9	9	x	x	x		Cattle, goat, human, sheep	Campania	5
9	10	9	7			x	x	Cattle, goat, sheep	Sicily	8
10	12	6	7	x	x	x	x	Goat, sheep	Calabria, Sicily	5
11	10	6	5	x	x	x		Goat, sheep	Calabria	5
12	9	6	7		x			Sheep	Sicily	5
13	17	5	4		x	x		Cattle, goat, sheep	Calabria	6
14	13	5	10	x	x			Goat, sheep	Sicily	5
15	8	5	7			x	x	Cattle, goat, sheep	Sicily	8
16	7	5	7	x	x	x		Goat, sheep	Sicily	7
17	5	4	2		x	x		Goat, sheep	Sicily	4
18	7	3	7		x	x	x	Cattle, goat, sheep	Sicily	3
19	8	2	7	x				Sheep	Sicily	1
20	6	2	4	x	x	x	x	Human, sheep	Abruzzi, Apulia	1
21	5	2	4			x	x	Cattle, goat, sheep	Sicily	1

The two largest clusters, C1 and C2, each containing approximately 10 % of the sequenced isolates, persisted over the entire studied period. While the prevalence of the C2 clone dramatically decreased after detection of the outbreak and implementation of control measures in 2015 and 2016, the number of brucellosis cases caused by the C1 clone has been increasing (Fig. 5a). C1 isolates were traced to 31 farms found in several locations in the east of Sicily and in the south of Calabria, and were obtained from sheep, goats and cattle. Interestingly, the majority of the clonal complexes contained strains that were isolated from more than one animal species, and three complexes also contained *B. melitensis* genomes obtained from human samples.

In several of the largest complexes, we found that the difference between pairs of the most genetically distant isolates exceeded the cluster cut-off of six core genes. This observation was true even for the strains isolated within the same year, suggesting that they likely were not directly related, but might have shared a recent common ancestor.

## Discussion

Brucellosis remains one of the most widespread zoonoses in the world despite implementation of surveillance, control and eradication campaigns at the national and international scale. In the EU, the disease is endemic in sheep and goats in some of the southern member states, which annually record the highest number of human brucellosis cases [25]⁠. Disease incidence in humans is further increased by infections acquired outside of Europe, mainly from the Middle East and Africa [38, 52, 53]. Between 2011 and 2018, the European Centre for Disease Prevention and Control (ECDC) reported 3645 human cases of brucellosis in the EU, 30 % of which were diagnosed in Italy (dataset provided by the ECDC based on data provided by the World Health Organization and the Ministries of Health from the affected countries) [54]. In our study, we used WGS tools to analyse a large set of isolates collected during the same time period and provide a comprehensive picture of the epidemiology, genetic diversity and evolutionary history of *B. melitensis* in Italy.

To date, in the studies that used WGS to characterize the global population of *B. melitensis *, the West Mediterranean lineage was under-represented due to a lack of sequenced isolates [16, 53, 55]⁠. In our work, we included recently published WGS data from Italy, as well as an additional 339 genomes; thus, expanding the phylogeny and providing a comprehensive analysis of the West Mediterranean lineage. The SNP-based phylogenetic analysis and subsequent BAPS group assignments divided this lineage into two diverse clades. Isolates from both populations were found to circulate in Sicily and Calabria, but only BAPS 4 strains were responsible for outbreaks in the central and northern regions. The factors behind geographical restriction of BAPS 5 and more widespread distribution of the other clade are not clear; however, it is possible that the BAPS 4 isolates were simply more numerous and, therefore, more likely to be transferred to other regions by trade.

Studies on the geographical partitioning of goat species revealed the presence of genetic groups related to the following geographical areas: the East, Central and West Mediterranean, and Central/Northern Europe [56, 57]. The genetic distances suggested that goats were first domesticated around 10 500 years ago in the Fertile Crescent, from where they spread to Europe along with Neolithic human populations [58]. Similar observations have been made for sheep [59]. Our phylogenetic analysis of *B. melitensis* follows similar phylogeographical patterns. According to our temporal analysis, the West Mediterranean lineage was the first to diverge from the original East Mediterranean lineage more than 5 500 years ago, which is similar in timing to previous estimates [16]⁠. Our findings for *B. melitensis* population divergence coincide with the timing of the Neolithic expansion, suggesting that the bacterium likely arrived in the central Mediterranean with infected sheep or goat populations. Then, the West Mediterranean lineage evolved in the region within its own species of ruminants. Although there is no archaeological evidence for the exact date of introduction of *B. melitensis* to Italy, we suspect that it happened 5 000 years ago. Osteological evidence supports likely brucellosis in people from the archaeological sites of Pompei and Herculaneum in 79 BCE [60]⁠. Furthermore, Kay and colleagues demonstrated the presence of the West Mediterranean lineage in Italy (Sardinia) using a metagenomics approach on a skeleton dated from the 1300s [61]⁠.

In our study, we observed two major subsequent divisions of the West Mediterranean lineage that occurred around the year 600 CE, the first splitting it into two subclades corresponding to BAPS 4 and BAPS 5, and the second that further divided BAPS 4. Since both populations are now prevalent in Sicily, at least two separate introduction events (or introduction and re-introduction) appear to have occurred in this region. In the BAPS 4 population, we observed two subpopulations. One of these was entirely composed of Italian isolates, while the other showed Italian isolates from the mainland along with French strains. The branches of the latter were long, suggesting an extended time for evolution within the cluster, partially explained by the geographical distribution of the sampled animals. Interestingly, we found that the Italian *B. melitensis* strain most closely related to the strain from France had been isolated from an alpine ibex from the Gran Paradiso National Park. The close localization of this park to the Bargy area in France suggests that this genotype is probably well established in France; however, further studies are required to understand the genetic diversity of *B. melitensis* found in this region. Within the BAPS 5 population, we identified two branches corresponding to strains from Egypt, Morocco and Eritrea that diverged from the Italian strains approximately 800–1000 years ago. The history of brucellosis spread in the Mediterranean basin could be explained by the trade connections between the southern Italian regions, Sicily in particular, and the Arabs and Berbers from Africa. While the West Mediterranean lineage may have been introduced to Italy in the Neolithic period, the spread of this lineage in Italy was probably associated with human movements. A strong trade connection between Italy and North Africa lasted from the time of the Roman Empire to the Middle Ages, and continued in the last century, in the period of modern colonialism. Commercial activities between both regions could, therefore, explain the history of brucellosis introductions between Africa and the Northern Mediterranean region [62]. At present, a limited number of strains from Africa and the lack of *B. melitensis* genomes from other European countries within the Mediterranean region prevents us from deciphering the full history of ovine and caprine brucellosis in this region.

Core-genome analysis of the Italian *B. melitensis* population identified 21 complexes of closely related isolates, several of which had been circulating in Italy for at least 8 years. Our data demonstrate that the C1 cluster has been steadily expanding, despite an aggressive eradication campaign. Moreover, unlike in the highly clonal C2, the genetic distances between individual isolates in C1 could be as high as 17 core genes. Similar genetic distances were also found for another large complex, C4, which was detected between 2013 and 2017 in four different regions. These data would suggest that there are still gaps in the brucellosis detection system or in the process of elimination of the infected animals, leading to some strains remaining undetected for many years. These strains then persist in the animal populations and transmit to new farms when different flocks are grazing in the shared pastures or through the trading activities; thus, hindering the elimination of *B. melitensis* from Italy.

The presence of a long branch within the BAPS 4 population, corresponding to a single strain isolated from a water buffalo, raises questions about the origins of the animal or the bacterial strain. It is possible that this isolate was derived from a genetic type that has now been largely eliminated due to the previous brucellosis eradication efforts. In fact, water buffalo in Italy are more commonly infected with *B. abortus* and the numbers of buffalo positive with *B. melitensis* have been steadily decreasing in the recent years [6, 32]. Analysis of genome sequences of *B. melitensis* strains circulating in Italy in the more distant past would be necessary in order to provide more information on the dynamics of emergence and extinction of specific lineages over time.

While using WGS in epidemiology and traceback of brucellosis cases has become a common practice, in particular in larger laboratories and reference centres, other methods such as biotyping are still recommended by the OIE and are, therefore, routinely performed. Here, the biotyping analysis showed that the Italian isolates of *B. melitensis* belong to biovar 3, but this biovar was also found in strains from the African and East Mediterranean lineages and, therefore, did not provide any additional epidemiological value for brucellosis investigations in Italy. MLST showed higher genotyping resolution than biotyping, as MLST-9 STs 11, 43, 88 and 91 were unique to the West Mediterranean clade and, therefore. could give an indication of the lineage. However, this typing method was not sufficient to distinguish between individual subclades.

WGS analysis, including cgMLST and whole-genome SNP typing, has superior genotyping resolution and is particularly well suited to large-scale epidemiological studies and disease surveillance monitoring when sufficient funding is available. However, it is important to note that in the case of insufficient sampling, or when few quality sequences are available, genetic linkage analyses might be misleading and genetically related sequences may not pass the cluster inclusion threshold. In our study, we included 441 Italian strains of *B. melitensis *; however, the actual number of positive animals between years 2011 and 2018 was several times higher [34]. Thus, it is possible that some connections between isolates or clusters were not sampled.

Ovine and caprine brucellosis has been a recognized problem in Italy for many decades. Despite the successful elimination of the disease in several northern regions of the country, the disease remains highly prevalent in Sicily and Calabria, affecting livestock production markets, limiting the live-animal trade and posing a lasting threat to human health. Moreover, the persistence of brucellosis in southern regions presents a continuous risk of spillover to the ‘officially *B. melitensis *-free territories’. While the geography of affected regions and the farming practices remain major factors in the propagation of the disease, the expansion of some of the genetic *B. melitensis* complexes in recent years suggests the current surveillance and eradication programme is not effective enough for the complete elimination of the ovine and caprine brucellosis. An in-depth study of the factors that are involved in disease transmission and limit the effectiveness of control campaigns would be vital for development of a successful eradication plan in *B. melitensis* hotspots in Italy.

## Supplementary Data

Supplementary material 1
